# Dynamics of pH Oscillators in Continuous Stirred Tanks in Series

**DOI:** 10.1002/cphc.202400610

**Published:** 2024-10-28

**Authors:** Csenge Galanics, István Szalai

**Affiliations:** ^1^ Eötvös Loránd University Institute of Chemistry Pázmány Péter sétány 1/A Budapest H-1117 Hungary

**Keywords:** nonlinear dynamics, multistability, oscillations, flow chemistry, autocatalysis

## Abstract

Complex reaction networks with positive and negative feedback can produce diverse nonlinear phenomena in open reactors, such as multistability and oscillations. pH oscillators driven by hydrogen or hydroxide autocatalytic processes show sustained oscillations in continuously stirred tank reactors (CSTR) but only a sharp pH switch in batch. Here, we present a numerical study on the dynamics of pH oscillators in a series of CSTRs. We show a critical residence time under which bistability and above which oscillations develop. The dynamics of the CSTR cascade show the cross‐shaped phase diagram of nonlinear activatory inhibitory systems. In the domain of oscillations, one reactor starts to oscillate autonomously and induces forced complex oscillations in the following tanks with damped amplitudes. These results, with their practical implications, may contribute to understanding the recent experimental observations of nonlinear phenomena in the presence of a residence time ramp and inspire further research in this area.

## Graphical abstract

A numerical study on the dynamics of pH oscillators in a series of CSTRs shows the emergence of bistability and oscillations. Within the oscillatory domain, one reactor oscillates independently, leading to forced complex oscillations in the subsequent tanks with decreasing amplitudes. A cascade of CSTRs can be an effective tool for studying the nonlinear dynamics of complex reaction networks.

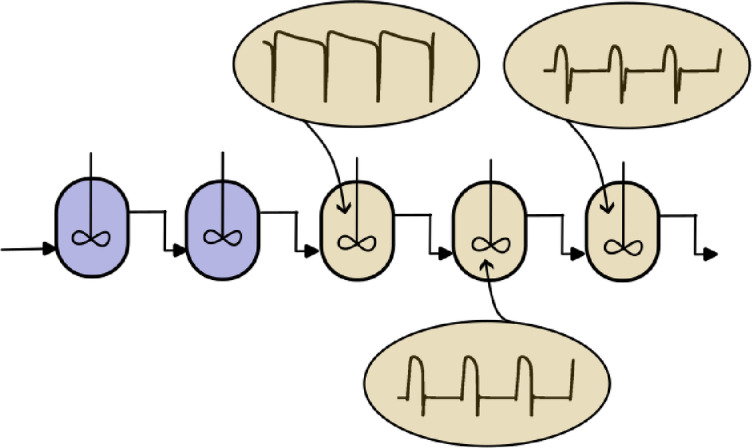

## Introduction

The mutual interaction of hydrodynamic flow and autocatalytic networks may open ways for the development of various dynamical phenomena, like fingering,^[1]^ convective dissolution,^[2]^ flow‐distributed oscillations,^[3]^ flow and diffusion‐distributed structures,[^4^, ^5^] and flow driven precipitation.^[6]^ It is also often assumed that far‐from‐equilibrium conditions provided by hydrothermal vents may support the autocatalytic synthesis of the organic compounds relevant to life's chemistry.^[7]^ Recently, flow reactors have become a favorable method in synthetic chemistry, even at the industrial level, especially when specific supramolecular structures are required.[^8^, ^9^, ^10^] Theoretical and experimental studies of autocatalytic reaction‐diffusion front in laminar flow revealed that the flow's direction strongly determines the front's shape and stability.[^11^, ^12^, ^13^, ^14^] When the flow and the chemical reaction front are unidirectional, the front velocities exceed the sum of that of the planar front in the absence of flow and the average flow velocity. In another case, when the direction of the flow and the propagation of the chemical front are opposite cusp‐shape front may develop that can even be stationary. Numerical simulations also predicted that oscillatory fronts may appear for strong enough flow velocities.^[15]^ The theoretical study of the dynamics of a one‐dimensional reaction‐diffusion‐advection system by using the Brusselator model revealed the appearance of diverse spatio‐temporal dynamics due to the presence of Hopf and Turing instabilities.^[16]^

Our recent experimental observations demonstrated that pH autocatalytic reaction networks may show bistability, excitability, and oscillations in a tubular flow reactor.^[17]^ The different dynamical states and phenomena may appear along the length of the tube due to the linear residence time ramp. Therefore, using tubular microreactors opens a unique experimental possibility to check the capability of chemical and biochemical reaction networks in producing nontrivial dynamical phenomena.

**Figure 1:**
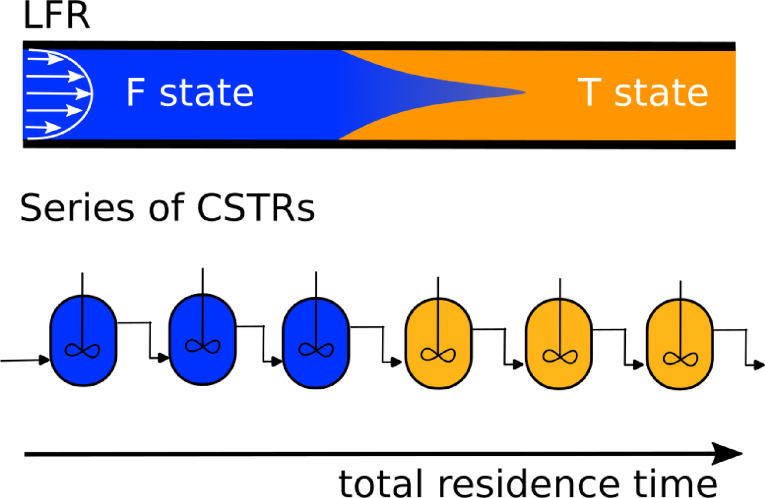
Sketches of a laminar flow reactor and a series of CSTRs.

The operation of a tubular reactor flow can be approximated as a series of coupled continuous‐stirred‐tank reactors (CSTRs).^[18]^ The common feature of these two types of reactors is the presence of the gradient of the total residence time (Figure 1). However, in a laminar flow reactor, the parabolic flow rate profile and the diffusive mixing can also play a role in the observed phenomena. These effects can not be accounted for in a series of CSTR models.

The following equation describes a series of *N* CSTRs:

(1) $$\frac{dc_{i}}{dt}=f_{i}\left(c_{i}\right)+\frac{1}{\tau }\left(c_{i-1}-c_{i}\right)$$

where, τ is the mean residence time of a reactor in the cascade, i=1..N denotes the serial number of the CSTR, $c_{i}$ is the concentration of chemicals in the *i*th CSTR, and $c_{0}$ is the input feed concentration of a chemicals and $f_{i}$ is the reaction terms *i*th CSTR. The mean residence time of a reactor in the cascade is defined as $\tau =\frac{V}{v}$ . Here, *V* is the volume of a CSTR, and *v* is the volumetric flow rate. We assume that the volume is the same for all CSTRs. The first CSTR has an input of fresh reactants ($c_{0}$ ), and the output of it acts as an input for the second CSTR. Each CSTR receives input from the previous one and provides input for the next one. Varma has shown that reactions that produce bistability in a single CSTR (with two stable and one unstable steady states), in a series of CSTRs with *N* reactors, have up to $2^{N+1}-1$ steady states, of which only *N*+1 are stable.^[19]^ The dynamics of a series of CSTRs were analyzed later using the theory of sequential bifurcation problems.^[20]^ Svoronos and coworkers have shown that oscillations can arise in the second tank when the first is in a steady state.^[21]^

This paper aims to characterize the nonlinear dynamics of a series of CSTRs with a reaction network capable of showing bistability and oscillations in a single CSTR. However, this reaction shows only a clock‐type behavior in a batch reactor. We used the Rabai model of pH oscillator, a general model of hydrogen ion autocatalytic reactions with negative feedback.^[22]^

In this model, B stands for an oxidant, A^−^ and HA are the unprotonated and the protonated forms of a weak acid, which are oxidized to the unprotonated form of a strong acid, C denotes a second reductant, and P and Q are products.

(R1) $$A^{-}+H^{+}\overset{⇀}{↽}\mathrm{HA}$$

(R2)

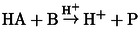

(R3) $$C+B+H^{+}\to Q$$

(R4) $$H^{+}+{\mathrm{OH}}^{-}\overset{⇀}{↽}H_{2}O$$

**Figure 2:**
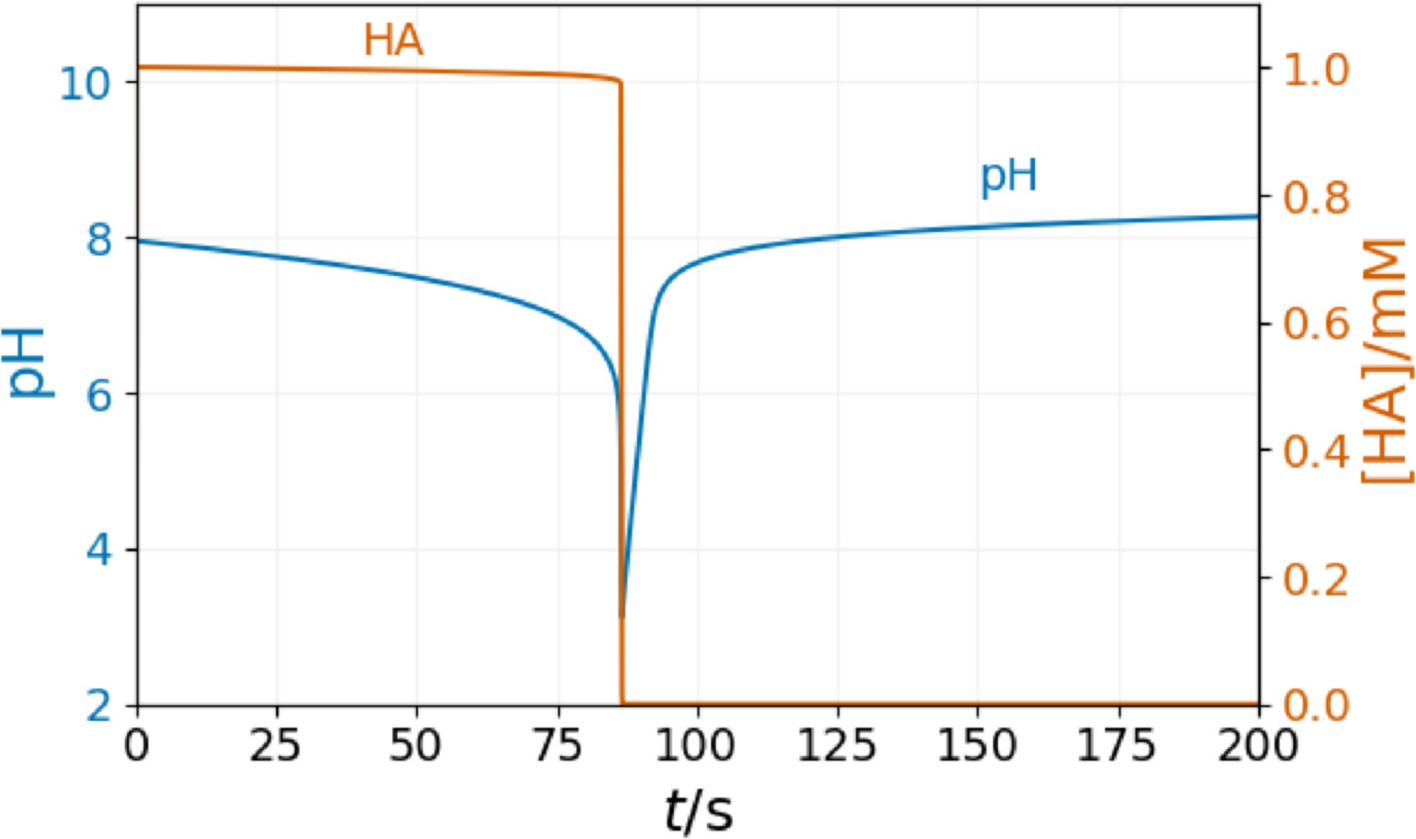
Batch behavior of the Rabai model at [B]_0_=15 mM, ${\left[A^{-}\right]}_{0}$ =10 mM, [C]_0_=5 mM, [H^+^]_0_=1 mM.

Reaction (R2) provides positive feedback as its kinetics is autocatalytic for H^+^. Reaction (R3) represents the negative feedback introduced by the presence of reactant C. This model can describe the core chemistry of most of the reactions used in the laminar flow experiments.^[17]^ In a batch simulation, a sharp acidic peak appears as it is shown in Figure 2. This model shows bistability and oscillations only in simulations corresponding to CSTR.[^22^, ^23^]

Here, we numerically explore the dynamics of the Rabai model in a series of CSTRs under conditions at which the compositions of the contents of the first few reactors correspond to the unreacted state. These simulations can reveal the effect of a gradient on the total residence time.

## Results and Discussion

**Figure 3:**
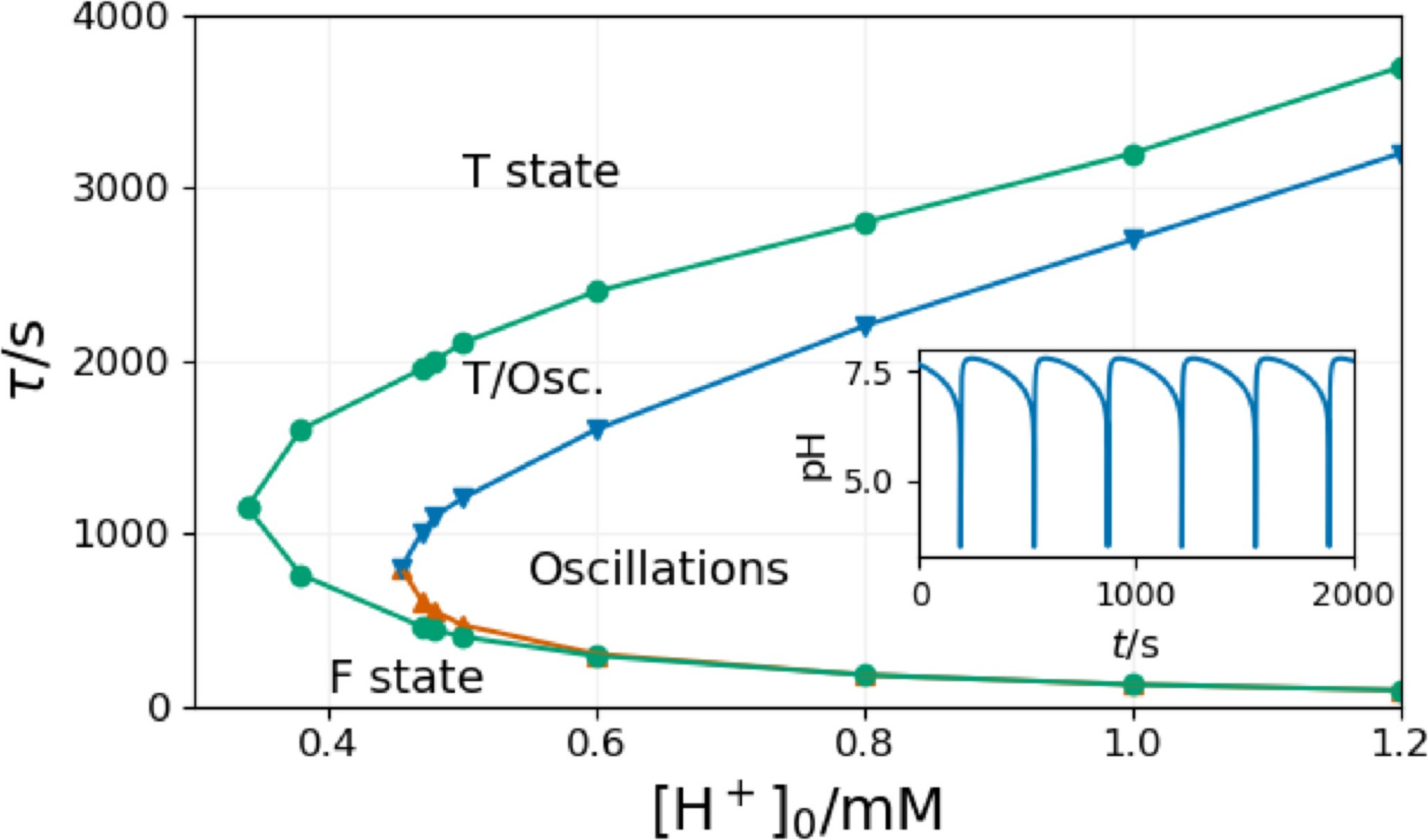
Nonequilibrium phase diagram of the Rabai model in a single CSTR at [B]_0_=15 mM, [A^−^]_0_=10 mM, [C]_0_=5 mM. Up and down triangles and bullets denote the stability limits of the F, T, and oscillatory states. The inset shows a typical oscillatory curve.

Let us first recall the dynamics of the Rabai model in a single CSTR.^[22]^ At the selected input feed concentration, the model shows oscillatory dynamics in various parameters. We explored the dynamics of the system in the field τ and ${\left[H^{+}\right]}_{0}$ parameters (Figure 3). At low values of τ , the extent of the autocatalytic reaction (R2) is low. Thus, the composition of the content of the CSTR is close to that of the input feed. It is the unreacted (F) state of the CSTR content. Above a critical ${\left[H^{+}\right]}_{0}$ , the F state becomes unstable by increasing the τ , and sustained oscillations appear. Further increasing τ , above a critical value, oscillations stop, and the content of the CSTR settles on a stationary state at which the extent of the autocatalytic reaction (R2) is high. This stationary state is called the T (“Thermodynamic”) state. In a range of parameters, the stability domain of the stationary F and T states overlap with that of the oscillatory state. This is the sign of subcrtitical Hopf bifurcation.

To describe the dynamics of the series of CSTRs, we use τ , the residence time of the CSTRs, as a control parameter. In order to reflect the experimental observations made in a tubular flow reactor,^[17]^ we used conditions at which at least the content of the first few reactors of the series of CSTRs was kept on the F state.

**Figure 4:**
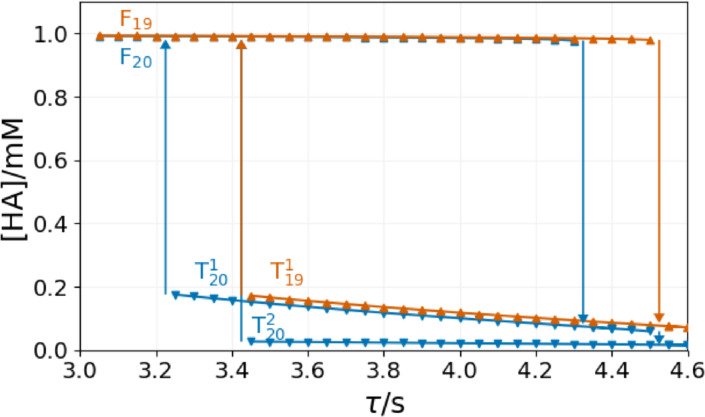
Bistability in the Rabai model in a series of CSTRs at [B]_0_=15 mM, [A^−^]_0_=10 mM, [C]_0_=5 mM, [H^+^]_0_=1 mM. Up and down triangles denote the stability limits of the F and T states.

In the numerical simulations performed with different numbers (*N*) of CSTRs below a critical value of τ , the content of all CSTRs is on the F state. Here, we select the 20^th^ reactor to represent the general aspects of the system's dynamics. In Figure 4, we start at τ =3 s where the content of 20^th^ CSTR is on the F state (F_20_). This state is stable up to τ =4.3 s, where the chemical composition of the content of the 20^th^ CSTR suddenly switches to a T state ($T_{20}^{1}$ ). At this point, the content of the 19^th^ CSTR is still in the F state (F_19_). Now, by decreasing τ the $T_{20}^{1}$ state is stable until τ =3.25 s, below it the content of the 20^th^ CSTR switches back to F_20_ state. It means a bistability exists between F_20_ and $T_{20}^{1}$ states in the range of 3.25<τ<4.3 . By increasing τ up 4.5 s, the F state of 19^th^ CSTR content becomes unstable and switches to a T state ($T_{19}^{1}$ ). At the same time, as the input of the 20^th^ reactor has changed, the state of the content must change, too. The content of the 20^th^ reactor switches from $T_{20}^{1}$ to $T_{20}^{2}$ state. Comparing the $T_{20}^{1}$ to $T_{20}^{2}$ state, the content's composition and stability range in τ differ. When the $T_{19}^{1}$ becomes unstable at τ =3.5 s and the content of the 19^th^ reactor switches back to the F state, the content of the 20^th^ reactor also switches from $T_{20}^{2}$ to the F state. Our numerical simulations did not show the transition from $T_{20}^{1}$ to $T_{20}^{2}$ state. In the range of 3.5<τ<4.3 the content of the 20^th^ reactor has three stable states: F_20_, $T_{20}^{1}$ and $T_{20}^{2}$ .

**Figure 5:**
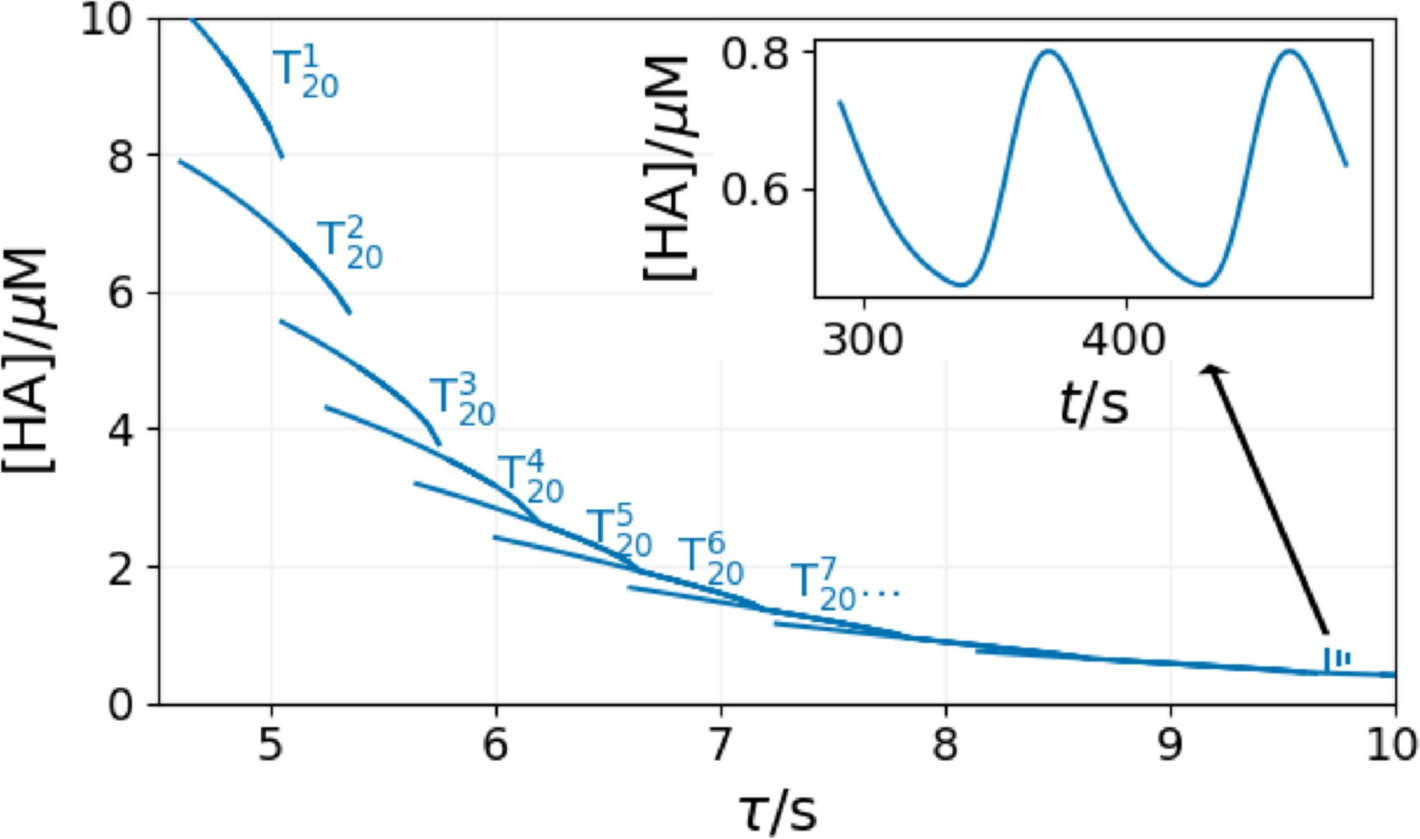
Multiplicity of the T state in the Rabai model in a series of CSTRs at [B]_0_=15 mM, [A^−^]_0_=10 mM, [C]_0_=5 mM, [H^+^]_0_=1 mM. The vertical lines around τ =9.8 s indicate the amplitude of oscillations.

The appearance of multiple T states corresponds to the result of the theoretical analysis.[^19^, ^20^] In Figure 5 the series of the T states of the 20^th^ reactor is presented. Above τ =4.3 s, only the different T states of the content of the 20^th^ reactor are stable. Bistability can be observed between the consecutive T states at some parameters. The difference between these T states vanishes with the increase of τ .

**Figure 6:**
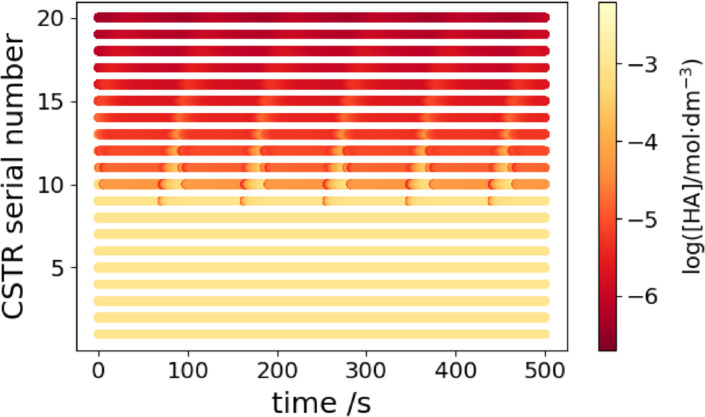
Time‐space plot of the dynamics at [B]_0_=15 mM, [A^−^]_0_=10 mM, [C]_0_=5 mM, [H^+^]_0_=1 mM and τ =9.7 s.

**Figure 7:**
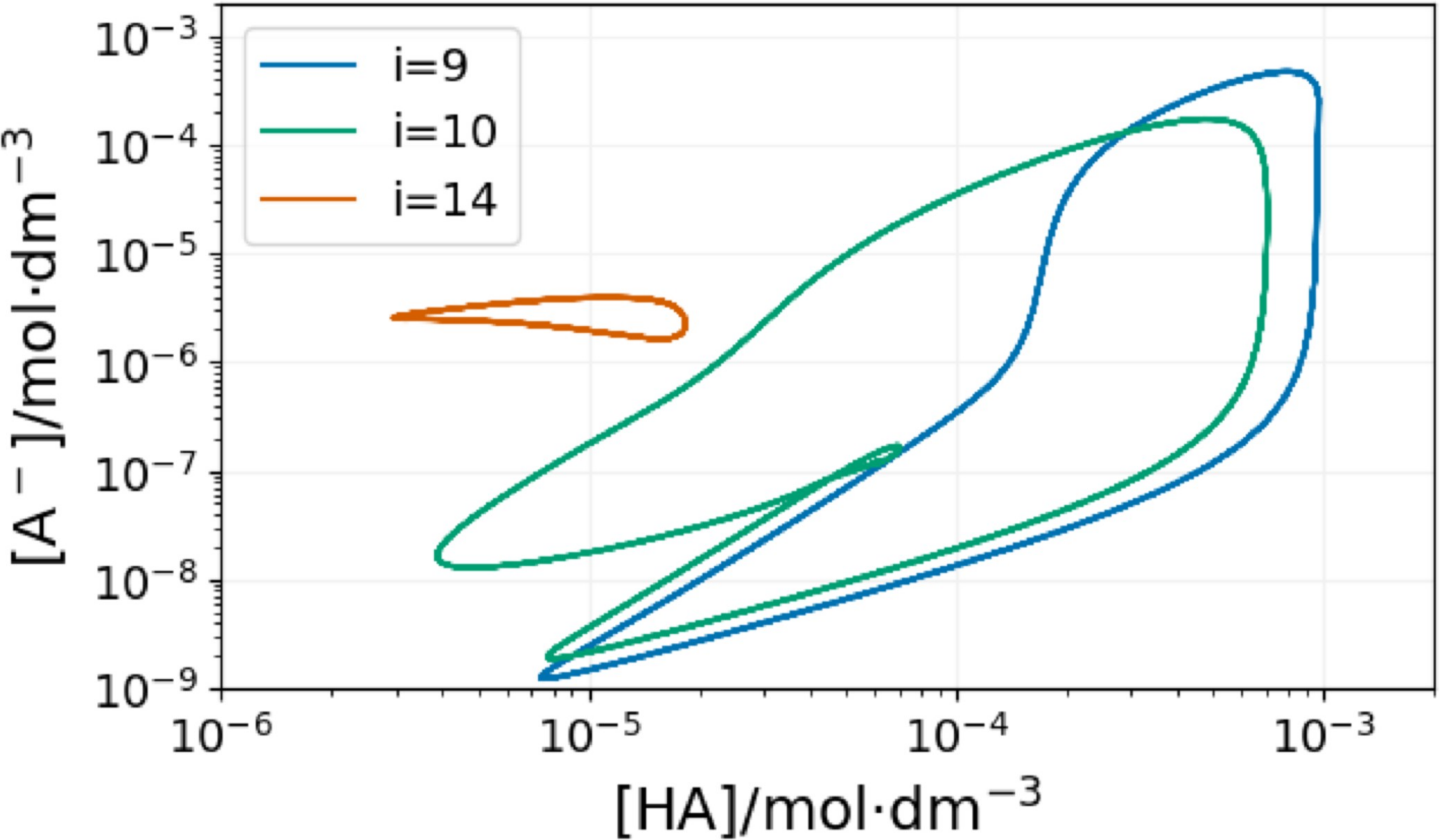
Projections of the limit cycle of the oscillations in the 9^th^ (blue line), 10^th^ (green line), and 14^th^ (orange line) reactors on the [A^−^]‐[[HA]]plane. The input feed concentrations are: [B]_0_=15 mM, [A^−^]_0_=10 mM, [C]_0_=5 mM, [H^+^]_0_=1 mM and τ =9.7 s.

At τ =9.7 s, the content of the 20^th^ reactor suddenly oscillates (Figure 5). In order to understand the origin of this phenomenon, the dynamics of the entire system must be checked. The space‐time plot in Figure 6 shows that the significant event happens in the 9^th^ reactor. The content of this reactor starts to oscillate while the previous ones are still in the F state. The following reactor is fed with the periodic outflow of the 9^th^ one. The interplay of the periodic feeding and the oscillatory kinetics in the 10^th^ CSTR results in complex oscillations, as shown in Figure 7.

Not only the 10^th^ CSTR, but all the reactors behind the spontaneously oscillating 9^th^ one receive a periodic inflow of chemicals. The complexity and amplitude of the resulting forced oscillations decrease from one reactor to the next.

**Figure 8:**
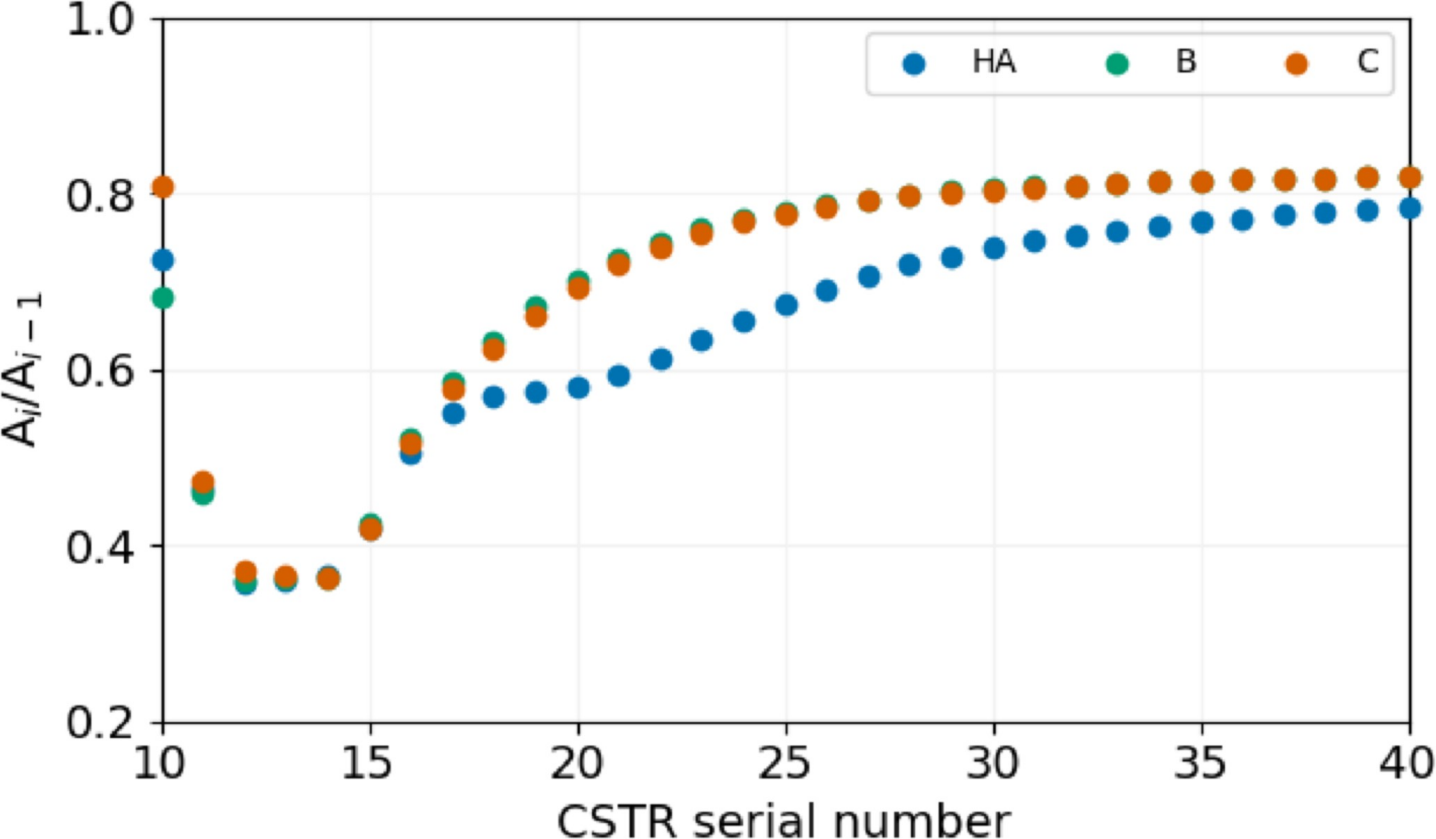
The ratio of the amplitude of oscillations in consecutive reactors at [B]_0_=15 mM, [A^−^]_0_=10 mM, [C]_0_=5 mM, [H^+^]_0_=1 mM and τ =9.7 s.

Figure 8 shows the ratio of the amplitude of the oscillations in the concentration of species HA, B, and C in two consecutive CSTRs (A_*i*_/A_*i*−1_). This relative amplitude first decreases in the case of all three species, but far from the spontaneously oscillating 9^th^ reactor by increasing reaches a constant value. The sharp decrease of the relative amplitude in the case of 10^th^–12^th^ CSTRs is the consequence of the sequential decrease of the input feed concentrations of the reactants.

The constant level of the relative amplitude from the 20^th^ CSTR is the signature of purely forced oscillation that forms without a significant contribution to the chemical reactions. The simplest case to describe forced oscillations in a series of CSTRs without reactions is applying a sinusoidal feed of a single chemical, that the following equation can describe:

(2) $$\frac{dc_{1}}{dt}=\frac{1}{\tau }\left(c_{0}+A_{0}\sin\left(\omega t\right)-c_{1}\right)$$

(3) $$\frac{dc_{i}}{dt}=\frac{1}{\tau }\left(c_{i-1}-c_{i}\right)$$

where, i=2..N .

The concentration of each CSTR shows sinusoidal oscillations with a damped amplitude and a phase shift ($\alpha_{i}$ ):

(3) $$c_{i}=c_{0}+\frac{A_{0}}{{\left(\omega^{2}\tau^{2}+1\right)}^{i/2}}\sin\left(\omega t+\alpha_{i}\right)$$

where, i=1..N . The ratio of the amplitude of the oscillations in two consecutive CSTRs is constant and can be described by he the following formula:

(5) $$\frac{A_{i}}{A_{i-1}}=\frac{1}{\sqrt{\omega^{2}\tau^{2}+1}}$$

This result indicates that when the relative amplitude of the oscillations in two consecutive CSTRs reaches a constant level in Figure 8, the contribution of chemical reactions to the oscillations is negligible in those CSTRs.

**Figure 9:**
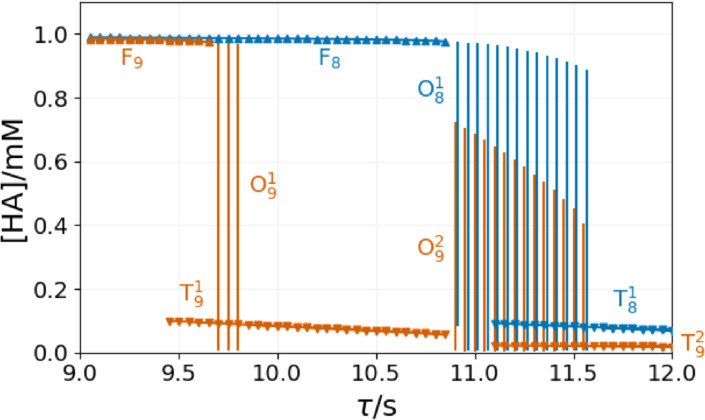
Bifurcation diagram of the Rabai model in a series of CSTRs at [B]_0_=15 mM, [A^−^]_0_=10 mM, [C]_0_=5 mM, [H^+^]_0_=1 mM. The symbols correspond to the stationary states, and vertical lines indicate the amplitude of the oscillatory states.

Figure 9 shows a bifurcation diagram of the content of the 9^th^ and the 8^th^ reactors. The stability domain of the oscillations around τ =9.7 s in 9^th^ reactor overlap with that of the T state. When the oscillations stop at τ =9.85 s the content of 9^th^ reactor settles on a T state, while the content of 8^th^ reactor is on the F state. In the range of 9.85s<τ<10.85s , the content of all CSTRs in the series is in a stationary state. The first eight reactors’ content is on an F state, and from 9^th^ reactor on a T state. The content of the 8^th^ reactor starts to oscillate at τ =10.9 s. In the range of 10.9s<τ<11.05s the oscillatory state is unique, but in the range of 11.05s<τ<11.55s there exists bistability between the oscillatory and the T state of 8^th^ reactor.

**Figure 10:**
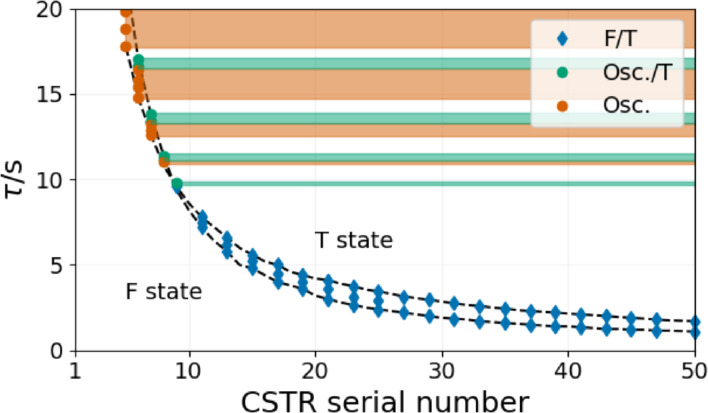
Nonequilibrium phase diagram of the Rabai model in a series of CSTRs at [B]_0_=15 mM, [A^−^]_0_=10 mM, [C]_0_=5 mM, [H^+^]_0_=1 mM. Blue diamonds correspond to bistability, green and orange dots correspond to bistability between the F and T states, bistability between the oscillatory and T states, and unique oscillatory states, respectively. The shaded domains indicate forced oscillations.

The overall dynamics of the series of a series of CSTRs is shown in Figure 10. The topology fits the general picture of open activatory‐inhibitory systems, often called a cross‐shaped phase diagram.^[24]^ Below, a critical value of τ (9.7 s) bistability between the stationary states (F and T) can be found in some members of the series of CSTRs. Above this critical value, oscillations appear in one CSTR, which induces oscillations in all following CSTRs. In between the oscillatory windows, quiescent regions develop. The width of these quiescent regions decreases with the increase of τ .

## Conclusions

The study of the dynamics of pH oscillators in a series of CSTRs was motivated by the experimental observation of bistability and oscillations in tubular flow reactors, either in the case where the flow was laminar or in the case of zigzag‐shaped channels designed for intense mixing.^[17]^ Numerical simulation of chemical reactions in a laminar flow, in general, requires the calculation of species concentrations along axial and radial directions, accounting for the interaction between transport processes and chemical kinetics. Here, we used the simpler CSTRs in a series model that exhibits pseudo plug flow characteristics to get an overall picture of the nonlinear dynamics. Therefore, our study does not count the effect of the laminar nature of the flow and the effect of diffusive mixing on the observed phenomena.

The presented results agree with previous theoretical studies that showed the appearance of multiple steady states and oscillations in a series of CSTRs.[^19^, ^20^, ^21^] However, our numerical study was not only focused on the dynamics of a few, typically two for theoretical tractability, linked reactors but on the overall dynamics of an extended series of CSTRs. The most exciting finding is the appearance of a cross‐shaped type phase diagram topology resembling a single CSTR's dynamics on the level of many CSTRs in a series. The formation of separated oscillatory windows manifests the discrete characteristics of the system. It is crucial to notice that oscillations develop above a critical value of τ . Only bistability can be observed at small τ values corresponding to the plug flow limit. In plug flow reactors, theoretically, thin “plugs” with uniform composition travel in the tubular reactor's axial direction, and the solution is perfectly mixed in the radial direction but not the axial one. The chemical system we used here is a prototype of the oscillatory reaction class that only shows oscillations in the presence of a continuous supplement of fresh reactants, e. g., in a CSTR. A plug flow does not fulfill this requirement; therefore, oscillations do not develop at those conditions.

CSTR cascades are suggested to provide safer, more efficient chemical production possibilities.^[25]^ Our numerical study points out that this type of reactor configuration can also be an effective tool for studying the nonlinear dynamics of complex reaction networks.

## Numerical Method

The rate laws of the reactions of the Rabai model are the following:

(v1) $$v_{1}=k_{1}\left[A^{-}\right]\left[H^{+}\right]-k_{-1}\left[\mathrm{HA}\right]$$

(v2) $$v_{2}=\left(k_{2}\left[H^{+}\right]+k_{2}^{'}\right)\left[\mathrm{HA}\right]\left[B\right]$$

(v3) $$v_{3}=k_{3}\left[C\right]\left[B\right]\left[H^{+}\right]$$

(v4) $$v_{4}=k_{4}\left[{\mathrm{OH}}^{-}\right]\left[H^{+}\right]-k_{-4}$$

The differential equations of the series of CSTRs model are the next:

(6) $$d{\left[H^{+}\right]}_{i}=-v_{1,i}+v_{2,i}-v_{3,i}-v_{4,i}\;\;\;+\frac{1}{\tau }\left({\left[H^{+}\right]}_{i-1}-{\left[H^{+}\right]}_{i}\right)$$

(7) $$d{\left[A^{-}\right]}_{i}=-v_{1,i}+\frac{1}{\tau }\left({\left[A^{-}\right]}_{i-1}-{\left[A^{-}\right]}_{i}\right)$$

(8) $$d{\left[\mathrm{HA}\right]}_{i}=v_{1,i}-v_{2,i}+\frac{1}{\tau }\left({\left[\mathrm{HA}\right]}_{i-1}-{\left[\mathrm{HA}\right]}_{i}\right)$$

(9) $$d{\left[B\right]}_{i}=-v_{2,i}-v_{3,i}+\frac{1}{\tau }\left({\left[B\right]}_{i-1}-{\left[B\right]}_{i}\right)$$

(10) $$d{\left[C\right]}_{i}=-v_{3,i}+\frac{1}{\tau }\left({\left[C\right]}_{i-1}-{\left[C\right]}_{i}\right)$$

(11) $$d{\left[{\mathrm{OH}}^{-}\right]}_{i}=-v_{4,i}+\frac{1}{\tau }\left({\left[{\mathrm{OH}}^{-}\right]}_{i-1}-{\left[{\mathrm{OH}}^{-}\right]}_{i}\right)$$

where, τ is the mean residence time of the reactors, i=1..N denotes the serial number of the CSTR, []_*i*_ is the concentration of a chemical in the *i*th CSTR, and []_0_ is the input feed concentration of a chemical.

Integrating the kinetic differential equations variable‐order method (LSODA) of the solve_ivp function of the NumPy and SciPy packages was used.^[26]^ The applied relative and absolute tolerances were 10^−8^ and 10^−15^, respectively. The simulations were started with empty CSTRs. The Python code used for the simulations is available under an Open Science Framework project.^[27]^

## Conflict of Interests

The authors declare no competing interests.

1

## Data Availability

The data that support the findings of this study are openly available in OSF at 10.17605/OSF.IO/VDQXJ, reference number 17605.
